# Shared enemies as prosocial tool: how to infer positive relationships from negative relationships in social networks

**DOI:** 10.1186/s40359-026-04098-0

**Published:** 2026-02-13

**Authors:** Yating Chen, Leping Wang, Qiang Zhou

**Affiliations:** 1 Department of Psychology, Wenzhou Medical University, Wenzhou, China; 2 Cixi Biomedical Research Institute, Wenzhou Medical University, Ningbo, China; 3 The Center for Geriatric Medicine, First Affiliated Hospital of Wenzhou Medical University, Wenzhou, China

**Keywords:** Social cognition, Relationship inference, Negative relationship, Intergroup relations, Social network

## Abstract

**Background:**

Social relationships are shaped by a complex interplay of interests and emotions, making negative interactions inevitable. Research on negative bias and loss aversion demonstrates that people are more sensitive to negative interactions, which often leads to social distancing. However, one study found that individuals with anxious attachment styles may actively seek partners who treat them poorly, suggesting that such relationships can strengthen bonds. But the cognitive mechanisms underlying how negative relationships can signal positive relational outcomes remain unclear. Through five experiments, we examine how shared negative relationships help individuals infer positive relationships and explore the underlying mechanisms.

**Methods:**

We conducted five pre-registered experiments (N = 1,085; *M*_age_ = 20.22, *SD* = 2.05) using hypothetical scenarios and social network methodologies. In Experiment 1, participants inferred interpersonal relationships from images depicting shared negative relationships. Building on this, Experiment 2 specifically focused on images of bidirectional shared negative relationships. Experiment 3 extended this paradigm to the intergroup level, where participants inferred relationships between groups based on shared negative relationships with a third-party. Experiment 4 refined this focus by examining in-group and out-group dynamics, asking participants to infer relationships from images of shared negative relationships between members of these distinct groups. Finally, Experiment 5 employed a simulated social network paradigm in which participants inferred relationships by learning and remembering social knowledge within an incomplete network, thus modeling real-world social inference under uncertainty.

**Results:**

Our results showed that humans can (a) infer positive relationships from shared negative ones and their quantity; (b) adjust these inferences by group size and contextual boundaries—shifting from resource-based inferring in ambiguous settings to identity-based mechanisms in well-defined groups; and (c) shape network representations from incomplete social knowledge, revealing human differences in cognitive mechanisms.

**Conclusions:**

While negative relationships can weaken social experiences, shared negative interactions can serve as prosocial tools for inferring positive relationships. Research shows that humans adjust their strategies based on relationship quantity and group structure, enabling them to build networks from incomplete social knowledge.

**Supplementary Information:**

The online version contains supplementary material available at 10.1186/s40359-026-04098-0.

## Introduction

In complex social environments, individuals have the unique ability to infer others’ interpersonal relationships by observing their interactions [1–3]. For example, we might notice that Joe and Lily frequently discuss the same books, leading to the inference that they are good friends. This relationship inference reflects the human tendency to associate similarities with the likelihood of strong relationships [4]. Such straightforward inference of a dichotomous relationship is supported by interdisciplinary evidence from the fields of psychology, sociology, and communication [5, 6], elucidating the foundations of social networks [7]. So, what mechanisms do humans rely on to efficiently accomplish this kind of social inference?

Similarity heuristics are a fundamental and pervasive mechanism for inferring relationships. In social networks, the assumption that “friends are similar” [8] leads to the formation of distinct groups whose members align in views, interests, and behaviors. These homophily-driven interactions reinforce similarities, as like-minded individuals influence each other’s decisions and actions [9, 10]. This process resembles associative learning, where repeated interactions enable individuals to learn about one another and adapt their behavior [11]. Moreover, both humans and animals can infer relationships indirectly by observing third-party connections [12], reflecting an over-perception of shared cues [13]. For instance, we commonly assume that two persons with a mutual friend will eventually become friends. Research shows that ternary closure has a greater impact than individual similarity [14]. Consequently, people tend to infer interpersonal relationships through shared relationships, which may represent a form of higher-order social similarity. While previous studies have emphasized the role of similarity and third-party connections in relationship inference, they often overlook other common relationship types within social networks, thereby limiting our comprehensive understanding of relationship inference.

The significance of social relationships and network diversity for physical and mental health, as well as social well-being, has been extensively documented [15]. A growing body of research emphasizes the importance of shared positive relationships (friends of friends) [16]. However, social networks are complex and encompass both positive and negative relationships. Negative relationships can harm health [17], exacerbate conflicts, disrupt group interactions [18], and intensify interethnic segregation [19]. Despite insights from numerous studies, critical questions regarding the cognitive mechanisms underlying friendship inferences in social contexts characterized by shared negative relationships (enemies of enemies) remain unresolved. Specifically, the question arises: when we learn that two people share a mutual dislike for someone, can we infer a positive relationship between them based on this shared negative relationship? Or does this shared negative relationship actually prevent us from making such positive inferences?

To understand the impact of shared negative relationships on relationship inference, it is crucial to place it within the complex social networks. An individual’s social connections are shaped not only by direct interactions but also by the number of shared contacts [20, 21]. Research has shown that the number of shared contacts within a network often exceeds expectations, and establishing friendships through indirect third-party connections is generally easier than anticipated [22]. Notably, the number of shared contacts tends to enhance the likelihood of friendship reference [23], especially in contexts involving shared positive or negative relationships. In other words, we may infer the existence of friendships between strangers based on the number of shared negative relationships.

Intergroup relations are a significant social dynamic in group interactions. Humans engage in various forms of interaction, ranging from pairs (such as couple relationships) to larger groups consisting of dozens of people (such as collaborative work) [24]. Group size affects social cognition and emotional responses, including individuals’ feelings of emotional security and threat [25], while also shaping people’s inferences about group status and competence through different cognitive mechanisms [26]; in turn, this affects the process of building social relationships. Other research has demonstrated a quantitative relationship between group size and individual preferences, as well as the brain regions that significantly contribute to social cognition [27]. Therefore, group size and the number of relationships may jointly influence relationship inferences, thereby shaping how individuals process complex knowledge within these social networks.

Interactions between individuals and groups shape the structure of social networks. Given limited cognitive resources, constructing cognitive maps based on social relationships enables individuals to overcome constraints in information processing, thereby simplifying complex social networks—an efficient cognitive strategy [28]. Cognitive maps not only provide a foundation for representing social networks but also serve as tools for understanding and executing abstract relational tasks, particularly in the domain of social knowledge [29, 30]. Simplified networks exhibit specific characteristics: each node (N) represents an individual’s social relationship, while edges (M) denote connections between these relationships. The length of edges between any two nodes follows a logarithmic relationship with network size [31, 32]. This logarithmic structure ensures connectivity in large networks while facilitating the accumulation of social capital—key resources gained through social interactions [33]. Constructing cognitive maps enables individuals to identify statistical dependencies among relationships [34, 35], thereby enhancing the speed and accuracy of relationship inference. This process is closely linked to similarity heuristics, as recognizing patterns and similarities in social relationships can significantly inform an individual's inferences and predictions about interpersonal dynamics. In summary, cognitive maps built upon social relationships can predict friendship between any two people, provided their social relationships are known.

**Fig. 1:**
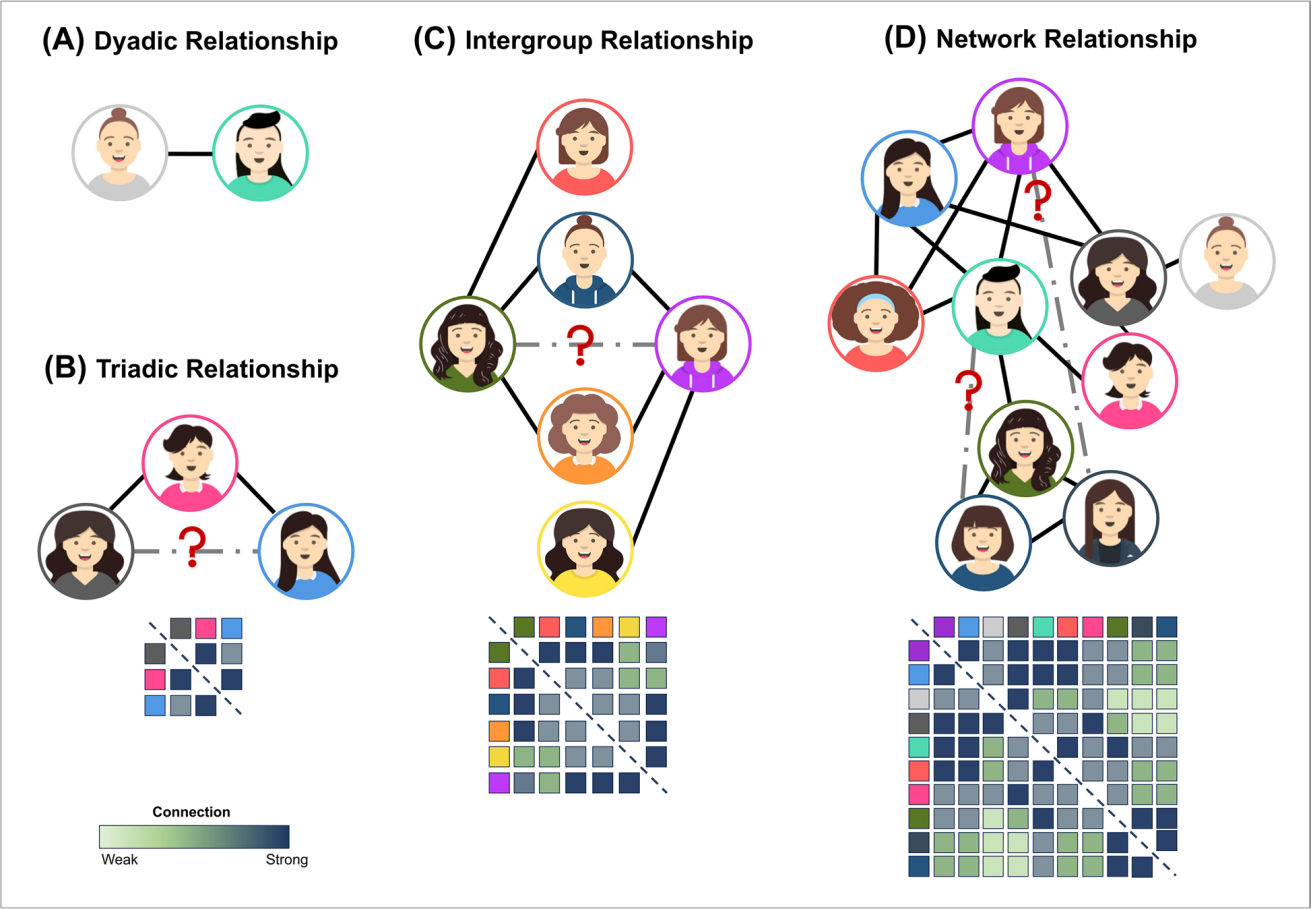
Theoretical framework of interpersonal relationships. Note. **A** Binary reasoning is the most basic form of inferring relationships. **B** Ternary closure emphasizes the formation of transfer relationships within social triads. **C** Tracking intergroup dynamics. **D** Social networks of interactions between individuals and groups integrate micro-level interactions in a comprehensive network of relational complexity

Humans possess the cognitive ability to infer unknown relationships within social networks through shared relationships. The present study aimed to examine whether individuals use shared negative relationships to infer interpersonal connections. Specifically, a question arises as to whether humans infer unknown relationships based on negative ones? Additionally, do the number of relationships and the size of the group influence relationship inference? Furthermore, we investigated the cognitive mechanisms underlying relationship inference in social networks. Thus, in Experiments 1 and 2, participants were required to identify shared negative relationships within groups to infer the relationship between two people. In contrast, experiments 3 and 4 necessitated that participants also consider the interactions among different groups. Finally, in Experiment 5, we simulated a real social network and assessed participants network representations on the basis of their cognition of shared relationships, as well as the cognitive mechanisms allowing them to make flexible inferences about unfamiliar relationships within the social network (Fig. 1).

### Experiment 1: The influence of shared negative relationships and group size on relationship inference

The similarity heuristic posits that when two people identify a shared enemy, they instinctively perceive a high degree of similarity in their social contexts, and this cognition promotes the establishment of alliances [36]. Within this framework, individuals exhibit heightened sensitivity to the quantity of shared relationships, rendering group size a critical factor in shaping relational inferences. Consequently, it is anticipated that participants will focus particularly on the number of negative relationships and utilize this knowledge to infer unknown relationships. Moreover, when the group size of a third-party mutual associate is small, the probability of a friendship forming between people A and B increases. This effect arises because larger groups tend to elevate individuals’ expectations regarding the number of shared relationships, and such heightened expectations may impede the relationship inference.

## Method

### Participants

The minimum sample size required for linear regression analysis was 129 participants in Experiment 1 (α = 0.05, f = 0.15, 1-β = 0.95), as calculated using G*Power 3.1.9.7. A total of 256 participants were recruited in experiment 1, but 15 participants who failed at least one pre-test and one post-test were excluded, resulting in a final sample of 241 participants included in the analyses (*M* = 20.35 years, *SD* = 1.74 years; 139 females, 102 males). This sample size provided 80% power to detect an effect size of r = 0.22 or greater in a linear multiple regression with 3 predictors, with a 5% false-positive rate. All participants were right-handed, had normal or corrected visual acuity, and did not report any color vision deficiencies, and all participants received 5 course credits as compensation for their involvement.

### Material and procedure

The face material is generated by Avatar Maker (chrome-extension://ofknlbikfofijlcjkfcihomkedmchfbn/app.html) to ensure that the face stimuli for experiments were distinct. Experiments 1–4 are adapted from Inferring Relations from Mutual Friends [23], shifting the focus from mutual friends to shared negative relationships. This experiment examines how shared negative relationships influence inferences about potential positive relationships (friendships) between two target people.

Experiment 1 utilized a 2 (group size: small vs. large) × 3 (shared negative relationships: few, medium, many) within-subjects design, with a controlled total of six negative relationships between the two targets to ensure that the observed effect was due to shared negative relationships. The levels of shared negative relationships were operationalized as one (few), three (medium), and five (many). Group sizes were defined as 12 members for small groups and 24 members for large groups. During the experiment, participants were presented with images illustrating two targets and their negative relationships with others within a third-party group, represented by unidirectional arrows indicating negativity. The procedure comprised three phases: pre-test, formal experiment, and post-test. In the pre-test phase, participants reported the number of negative relationships the target people had within the third-party group, serving to ensure comprehension of the task. In the formal experimental phase, participants viewed multiple images and were asked to estimate the likelihood of friendship between the two targets, rating this probability on a 7-point Likert scale (1 = completely unlikely, 7 = completely likely). Each image depicted people A and B alongside their negative relationships within the third-party group. The post-test phase included three questions: a single-choice item asking participants to classify the relationship between the two targets as friends, enemies, family members, or look-alikes, and two questions replicating the pre-test task of reporting the number of negative relationships for the target people. This phase aimed to assess participants’ understanding of shared negative relationships and inference criteria, ensuring accurate task comprehension and relationship inference.

## Result and discussion

**Fig. 2:**
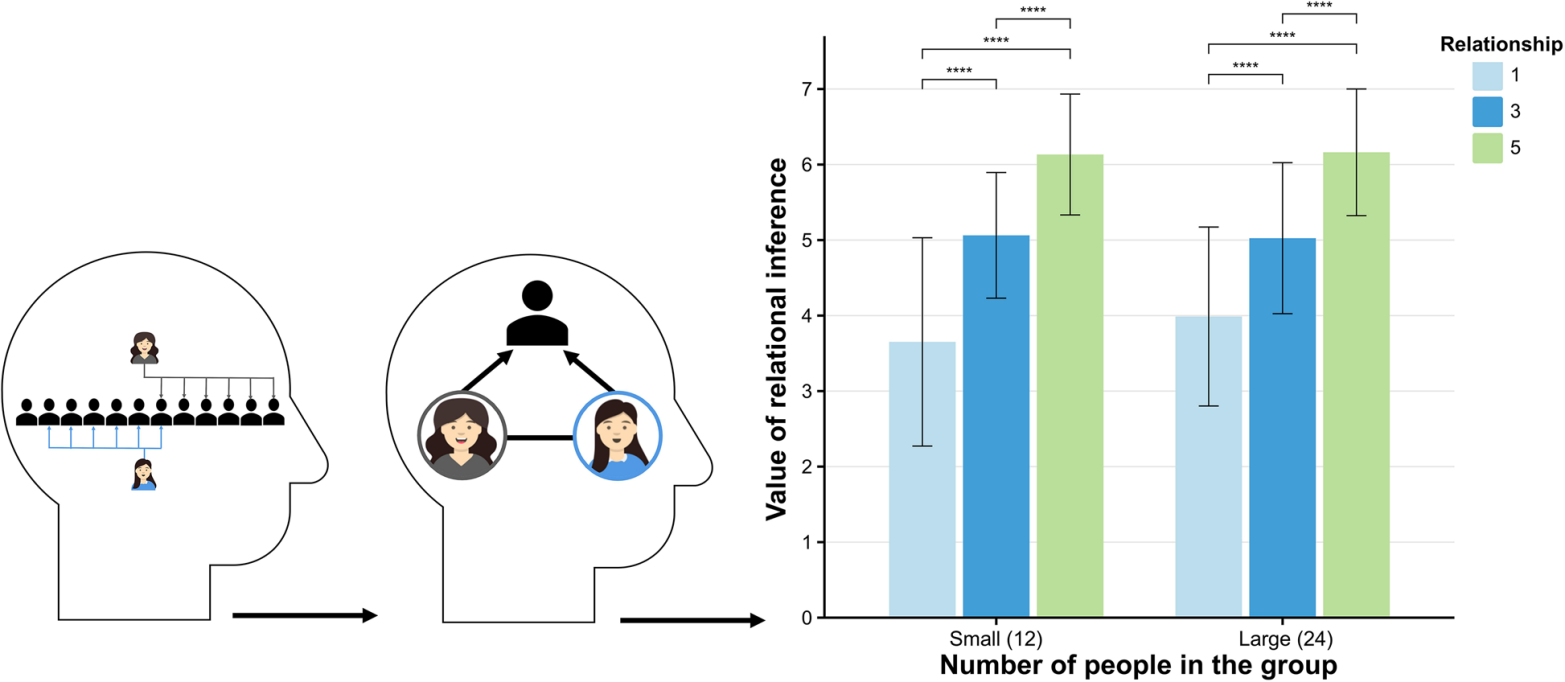
Inferring ternary closure in interpersonal relationships. Note. Statistical significance is indicated by an asterisk: *p <.05., **p <.01, ***p <.001, ****p <.0001

We employed the pre-registered linear generalized estimating equation (GEE) model for our analysis by R version 4.3.2, using group size and the number of relationships as predictor variables, inferred probability as a dependent variable (see Supplementary A). The results indicated that participants inferred a higher probability of friendships among two targets in a large group compared to a small group (*β* = 0.11, *SE* = 0.05, *z* = 2.34, *p* = 0.019). Additionally, participants perceived a higher probability of a two-person friendship when there were many shared relationships compared to only a few shared relationship (*β* = 2.33, *SE* = 0.80, *z* = 29.24, *p* < 0.001) and medium shared relationships (*β* = 1.10, *SE* = 0.46, *z* = 23.95, *p* < 0.001). This suggests that the greater the number of shared negative relationships, the more likely participants inferred that the two targets would form a friendship. The interaction between group size and shared negative relationships was significant (*β* = −0.006, *SE* = 0.002, *z* = −3.41, *p* < 0.001), with participants inferring that the probability of a two-person friendship was lowest in a small group with a few negative relationship (*β* = −0.31, *SE* = 0.08, *z* = −3.86, *p* < 0.001) (Fig. 2).

Experiment 1 tested several key hypotheses regarding negative relationships, emphasizing the significant roles of shared negative relationships and group size in relationship inference. Unexpectedly, larger group sizes were associated with a higher likelihood of participants inferring relationships. This finding contrasts with previous research on relationship inference based on shared friends [23], which indicated that smaller group sizes were linked to a higher probability of friendship. In contrast, the present study reveals a different pattern. We believe that this difference arises from the function of social capital, with larger organizations offering greater social capital, including resources and opportunities for new interactions [37, 38]. People may perceive that members of larger organizations possess more social capital, which increases the probability of positive associations while diminishing the impact of negative ones. This insight highlights the contrasting values of negative and positive relationships. The relationship between group size and the number of ties can be attributed to the fact that interactions among people in larger groups are more complex, and shared negative relationships can serve as a social tool to connect people. We will elaborate on this issue in the general discussion.

### Experiment 2: The influence of bidirectional shared negative relationships and group size on relationship inference

The fundamental nature of a relationship is inherently reciprocal: when Joe forms a connection with Lily, Lily correspondingly establishes a connection with Joe. Consequently, shared bidirectional negative relationships should function as a more robust indicator for inferring friendship. Consistent with the results of Experiment 1, we hypothesize that participants will particularly attend to the quantity of mutual negative relationships and utilize this knowledge to infer friendships. Additionally, the effect of group size remains significant. We anticipate that larger groups will elevate individuals’ expectations regarding the number of shared relationships, while an increase in group size may concurrently reduce the probability of friendship formation.

## Method

### Participants

The minimum sample size required for linear regression analysis was 129 participants in Experiment 2 (α = 0.05, f = 0.15, 1-β = 0.95), as calculated using G*Power 3.1.9.7. A total of 208 participants who did not participate in the previous experiment were recruited. 8 participants who failed at least one pre-test and at least one post-test was excluded, resulting in a final sample of 200 participants included in the analyses (*M* = 20.57 years, *SD* = 3.79 years; 113 females, 87 males). This sample size provided 80% power to detect an effect size of r = 0.24 or greater in a linear multiple regression with 3 predictors, with a 5% false-positive rate. All participants were right-handed, had normal or corrected vision with no reported color vision deficiencies, and received 5 course credits as compensation.

### Material and procedure

Experiment 2 utilized a 2 (group size: small vs. large) × 3 (shared bidirectional negative relationships: few, medium, many) within-subjects factorial design, with the total number of negative relationships between the two targets held constant at six. The levels of shared negative relationships were operationalized as one (few), three (medium), and five (many). Group size was defined as 12 members for small groups and 24 members for large groups. Bidirectional negative relationships were depicted using double-headed arrows. Participants received identical instructions and completed the pre-test phase consistent with Experiment 1. Subsequently, they engaged in six formal trials, each presenting an image illustrating two targets and their bidirectional negative relationships with a third-party group, followed by rating the likelihood of friendship inference on a 7-point Likert scale. The Experiment concluded with a post-test phase mirroring that of Experiment 1.

## Result and discussion

**Fig. 3:**
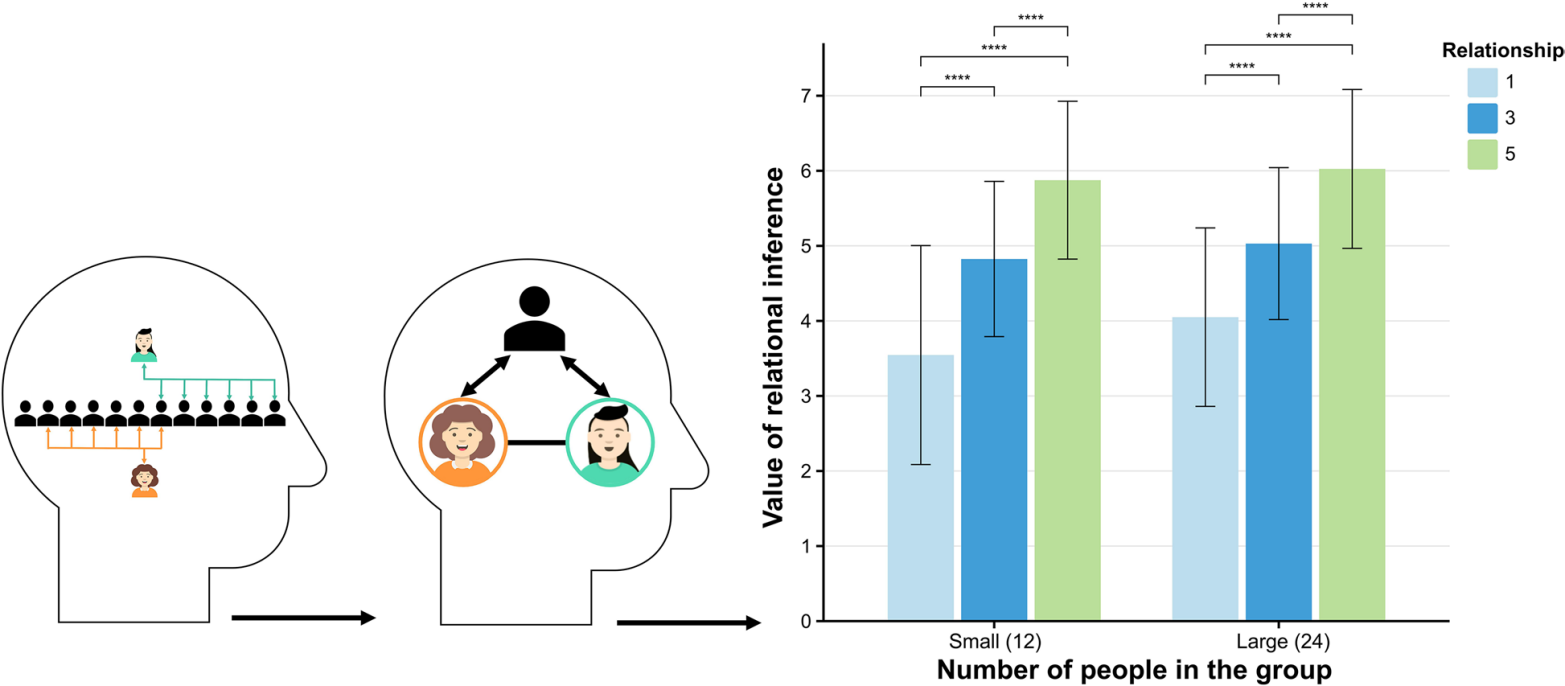
Inferring Bidirectional Ternary Closures in Interpersonal Relationships. Note. Statistical significance is indicated by an asterisk: *p <.05., **p <.01, ***p <.001, ****p <.0001

The results of the GEE analysis indicated that participants inferred a higher probability of friendship among two targets in a large group compared to a small group (*β* = 0.29, *SE* = 0.59, *z* = 4.88, *p* < 0.001). Participants also inferred a greater probability of friendship among two targets with many shared bidirectional negative relationships compared to those with only a few shared bidirectional negative relationship (*β* = 2.15, *SE* = 0.10, *z* = 22.53, *p* < 0.001) and medium shared bidirectional negative relationships (*β* = 1.02, *SE* = 0.06, *z* = 16.69, *p* < 0.001). This suggests that as the number of shared negative relationships increases, participants are more likely to infer that two targets have a friendship. The interaction between group size and shared negative relationships was significant (*β* = −0.007, *SE* = 0.003, *z* = 2.10, *p* = 0.036), with participants inferring that the probability of friendship was lowest among two targets with only a few negative relationship in a small group (*β* = −0.35, *SE* = 0.11, *z* = 3.22, *p* < 0.001) (Fig. 3).

Experiment 2 replicated the findings of Experiment 1, indicating that shared bidirectional relationships similarly influence relational inferences. This supports our hypothesis regarding the two-way reciprocity of relationships, suggesting that participants can acutely perceive negative dynamics in reciprocal interactions. As group size increases, the probability of participants inferring the establishment of relationships also strengthens. This phenomenon may be influenced by social capital; specifically, the characteristics of the group and the patterns of interaction may play a significant role in this process [39]. In the subsequent experiment, we examined the underlying premise of this explanation. We observed changes in participants’ relational inferences by manipulating group polarization and negative relationships within social networks.

### Experiment 3: The influence of shared negative relationships with third parties and intergroup size on relationship inference

Experiments 1 and 2 provide evidence that the similarity heuristic, grounded in shared negative relationships, effectively supports the inference of friendship between two targets. Nonetheless, in real-world settings, interpersonal relationships frequently extend across group boundaries, with interactions shaped not only by intra-group relations but also constrained by intergroup relations. The primary focus of Experiment 3 is to investigate whether the similarity heuristic can be applied across group boundaries. This experiment explores how participants infer relationships between two targets belonging to different groups who share a mutual connection with a third party. Specifically, it aims to (1) examine the influence of cross-group shared negative relationships and (2) extend the hypotheses derived from Experiments 1 and 2 by simulating intergroup relational structures to investigate how participants infer friendships within this context. Within this framework, we hypothesize that participants will continue to utilize the similarity heuristic to infer friendships across group lines. Additionally, we anticipate that group size will remain a significant factor, with larger groups potentially heightening participants’ expectations regarding the number of shared relationships, thereby influencing their friendship inferences.

## Method

### Participants

The minimum sample size required for linear regression analysis was 129 participants in Experiment 3 (α = 0.05, f = 0.15, 1-β = 0.95), as calculated using G*Power 3.1.9.7. A total of 257 participants who did not participate in the previous experiment were recruited. 13 participants who failed at least one pre-test and at least one post-test was excluded, resulting in a final sample of 244 participants included in the analyses (*M* = 20.57 years, *SD* = 2.09 years; 128 females, 116 males). This sample size provided 80% power to detect an effect size of r = 0.21 or greater in a linear multiple regression with 3 predictors, with a 5% false-positive rate. All participants were right-handed, had normal or corrected vision with no reported color vision deficiencies, and received 5 course credits as compensation.

### Material and procedure

Experiment 3 focuses on how participants infer relationships between two targets—when two targets belonging to different groups share a negative relationship with a third party—and examines whether this inference is influenced by group size. Utilizing a 2 (group size: small vs. large) × 3 (shared negative relationships: few, medium, many) within-subjects factorial design, the total number of negative relationships between the two focal people was held constant at six. The conditions for shared negative relationships were operationalized as few (one shared relationship), medium (three shared relationships), and many (five shared relationships). Small groups consisted of 12 participants, whereas large groups comprised 24 participants. Participants received standardized instructions and completed a pre-test phase consistent with that of Experiment 1. During the main experimental phase, participants completed six trials in which they viewed images depicting two targets and their shared negative relationships with a third party, situated within various third-party groups (e.g., negative relationships between Joe and Amy within the Soccer Group or between Lily and Amy within the Dance Group). After each image presentation, participants rated the likelihood of friendship between the two targets using a 7-point Likert scale. The experiment concluded with a post-test identical to that administered in Experiment 1.

## Result and discussion

**Fig. 4:**
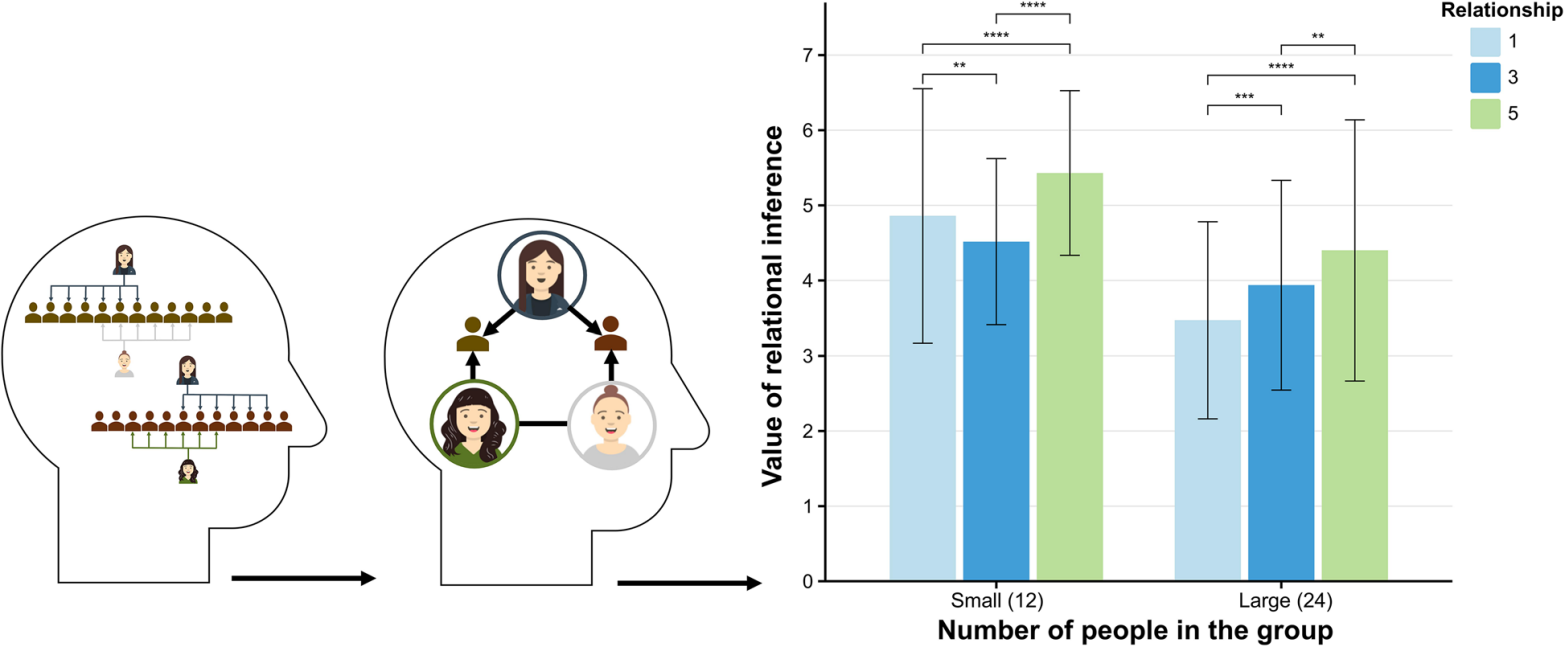
Tracking intergroup dynamics to infer relationships. Note. Statistical significance is indicated by an asterisk: *p <.05., **p <.01, ***p <.001, ****p <.0001

To assess whether relationship inferences are influenced by intergroup interactions, we conducted the pre-registered GEE analysis by R version 4.3.2 (see Supplementary A). The results indicated that participants inferred a higher probability of friendship among two targets in a small group compared to a large group (*β* = 1.00, *SE* = 0.94, *z* = 10.62, *p* < 0.001). Participants also inferred a higher probability of establishing a friendship among two targets with many negative relationships compared to those with only a few negative relationship (*β* = 0.75, *SE* = 0.86, *z* = 8.70, *p* < 0.001) and medium negative relationships (*β* = 0.69, *SE* = 0.63, *z* = 11.01, *p* < 0.001). This suggests that the greater the number of shared negative relationships, the more likely participants inferred that the two targets would establish a friendship. The interaction between group size and shared negative relationships was significant (*β* = 0.008, *SE* = 0.004, *z* = 1.97, *p* = 0.048), with participants inferring a higher probability of friendship when there was only a few negative relationship in a small group (*β* = 0.36, *SE* = 0.13, *z* = 2.79, *p* = 0.005). Conversely, in small groups, shared medium negative relationships significantly reduced the inferred friendship probability (*β* = −0.45, *SE* = 0.11, *z* = −4.13, *p* < 0.001) (Fig. 4).

The results of Experiment 3 align with the principles of social network theory, which posits that group size and the number of shared relationships among people are significant predictors of friendship formation [40, 41]. Notably, we found that when inferring interpersonal relationships between groups, smaller group sizes corresponded to a higher probability of relationship inference. This outcome diverges from the results observed in Experiments 1 and 2, which did not account for either intergroup or intragroup relationships. We posit that, in Experiments 1 and 2, participants based their inferences primarily on group size, presuming that larger groups were more prone to exhibit friendly ties within the context of shared negative relationships. However, the introduction of intergroup relationships in Experiment 3 revealed that participants’ estimations of friendship probabilities were influenced by intergroup dynamics, with smaller groups fostering closer friendships. This phenomenon may be attributed to the specificity of intergroup dynamics. In smaller groups, members tend to interact more frequently and develop closer bonds, thereby enhancing group cohesion [42]. Unexpectedly, participants inferred that two people with medium negative relationships within a small group had a lower probability of forming friendships compared to a few or many negative relationships. We propose that this may be attributed to the ambiguity of the information, which suggests that these shared relationships are insufficient for fostering stable relationships, particularly since the negative relationships themselves may be more destructive or isolating. Overall, even though our shared relationships belonged to two different groups, our co-contacts remained connected to an intermediary who facilitated relationship building.

### Experiment 4: The influence of shared negative relationships and in-group/out-group size on relationship inference

Experiment 3 identified the pivotal influence of group size and shared negative relationships on the inference of cross-group friendships. Extending this line of inquiry, Experiment 4 shifts the focus to within-group dynamics to examine how individuals infer friendships among ingroup members, considering both ingroup size and shared negative relationships. It was hypothesized that participants would base their inferences about interpersonal relationships on the size of the group to which people belonged. The objectives of Experiment 4 were twofold: first, to build upon the findings of Experiments 1 through 3 by further investigating the effect of group size on friendship inferences within ingroup contexts, thereby assessing the Experiment 3 finding that smaller groups facilitate friendship inferences; and second, to explore whether the existence of shared negative relationships between group members and external individuals (for example, Joe and Lily, both members of the soccer team, sharing a dislike toward certain members of the dance team) increases the propensity to infer a friendship between those group members.

## Method

### Participants

The minimum sample size required for linear regression analysis was 129 participants in Experiment 4 (α = 0.05, f = 0.15, 1-β = 0.95), as calculated using G*Power 3.1.9.7. A total of 225 participants who did not participate in the previous experiment were recruited. 14 participants who failed at least one pre-test and at least one post-test was excluded, resulting in a final sample of 211 participants included in the analyses (*M* = 20.10 years, *SD* = 1.53 years; 107 females, 104 males). This sample size provided 80% power to detect an effect size of r = 0.23 or greater in a linear multiple regression with 3 predictors, with a 5% false-positive rate. All participants were right-handed, had normal or corrected vision with no reported color vision deficiencies, and received 5 course credits as compensation.

### Material and procedure

In Experiment 4, the experimental design involved two targets within a single group who each maintained negative relationships with particular members of a separate group (specifically, Mark and Oli from the soccer team had negative relationships with members of the dance team). To enhance ecological validity and better represent group heterogeneity, experiment 4 substituted abstract silhouettes with cartoon portraits. The outgroup maintains 12 members. The Experiment utilized a 2 (ingroup size: small vs. large) × 3 (number of shared negative relationships: few, medium, many) within-subjects factorial design, with control procedures ensuring a total of six negative relationships between the two focal people. The ingroup sizes were operationalized as either a small group (6 members) or a large group (12 members). Participants received identical instructions and completed the same pretest phase as in Experiment 1, followed by six experimental trials. In each trial, participants were presented with images illustrating two targets within a group alongside their negative relationships with members of a third-party group, subsequently rating the probability of friendship formation on a 7-point Likert scale. Finally, participants completed the posttest identical to that administered in Experiment 1.

## Result and discussion

**Fig. 5:**
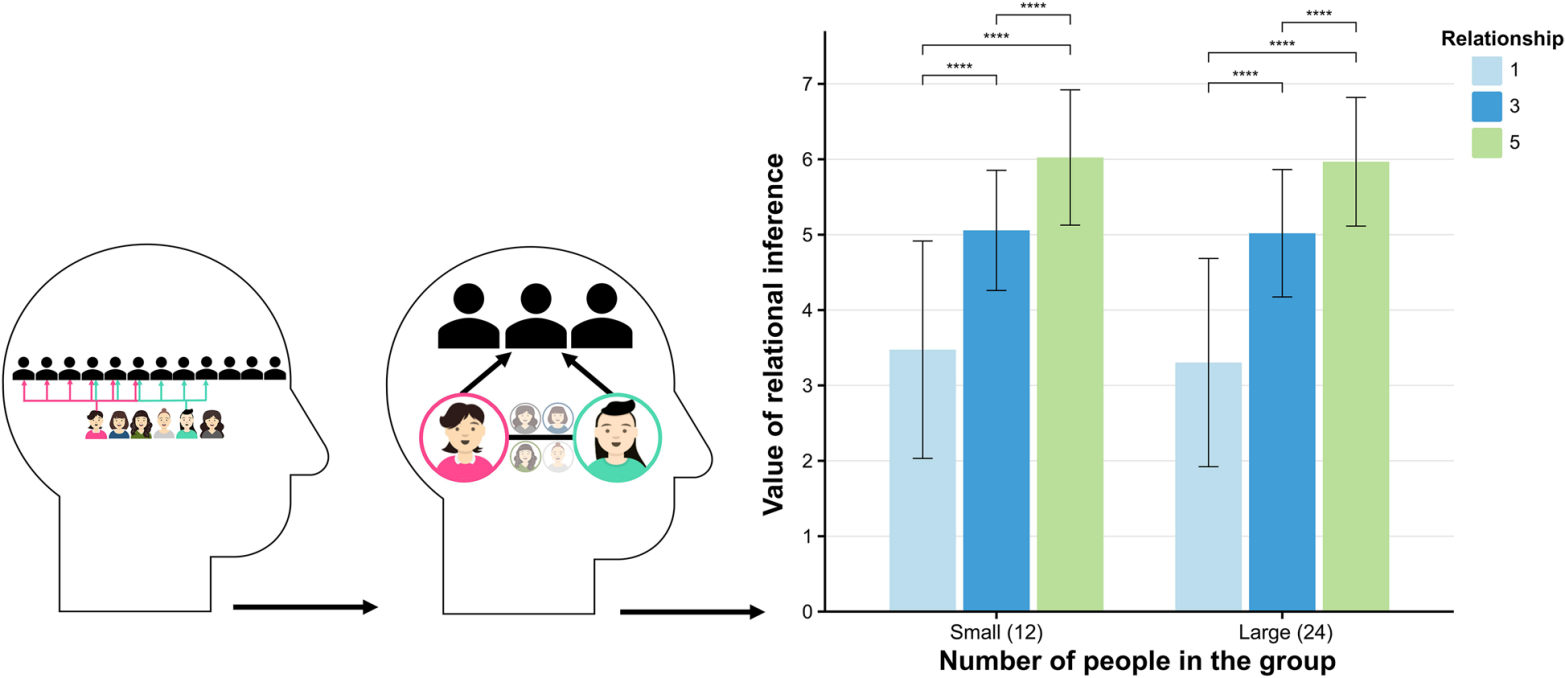
Tracking dynamics within and outside groups to infer relationships. Note. Statistical significance is indicated by an asterisk: *p <.05., **p <.01, ***p <.001, ****p <.0001

The results indicated that participants referred a higher probability of friendship between two targets in a small ingroup compared to a large ingroup (*β* = 0.09, *SE* = 0.04, *z* = 2.14, *p* = 0.033). Participants also inferred a greater probability of friendship for two targets with many negative relationships compared to those with only a few negative relationship (*β* = 2.61, *SE* = 1.00, *z* = 26.42, *p* < 0.001) and medium negative relationships (*β* = 0.96, *SE* = 0.44, *z* = 21.83, *p* < 0.001). This suggests that as the number of shared negative relationships increases, participants are more likely to infer that the two targets have a friendship (Fig. 5).

Experiment 4 supported several key hypotheses regarding relationship inference. First, smaller ingroup sizes were associated with a higher probability of participants inferring the establishment of relationships, thereby replicating the findings of Experiment 3. We propose that this phenomenon may be attributed to increased group cohesion, as participants tend to associate smaller groups with stronger interpersonal connections. Second, as predicted, the likelihood of participants inferring relationship establishment was greater when the number of shared relationships was higher. Third, the combined analyses revealed that the interaction between group size and shared relationships was not significant. This finding suggests that the effects of group size and co-relationships on friendship formation in real-life situations are not merely linearly cumulative; instead, they exhibit a logarithmic relationship [43]. To the best of our knowledge, Experiment 4 also offers some of the first evidence elucidating the mechanisms of social interaction, highlighting the validity and importance of co-relationships and group size in relationship inferences.

### Experiment 5: The impact of incomplete social knowledge on relationship inference in social networks and its cognitive mechanisms

Building upon the findings from Experiments 1 to 4—where shared negative relationships and group size significantly influenced relationship inference—Experiment 5 embedded this social knowledge within more ecologically valid, structured social networks. We simulated real-world network structures by incorporating social relationships within group characteristics (e.g., Joe is in the soccer club, Lily is in the choir club, and their friendship is based on a shared dislike of Alex). These social attributes partitioned the network into distinct subgroups, allowing us to explore relationship inference within more complex group contexts. Experiment 5 aimed to achieve three primary objectives. First, do participants utilize social knowledge (shared clubs and negative relationships) to infer relationships within social networks? Second, when making relationship inferences in social networks, what cognitive mechanisms (similarity heuristics, associative learning, and cognitive maps) do participants employ to shape their network representations, and are these mechanisms influenced by individual traits? Finally, does the acquisition of social knowledge inform cross-situational relationship inferences?

## Method

### Participants

We conducted a prior power analysis using G*Power 3.1.9.7 and determined that the minimum sample size required for a repeated-measures ANOVA with within-subjects design is 84 (α = 0.05, f = 0.2, 1-β = 0.95), based on the parameters established in previous experiment [44]. A total of 139 participants, who had not participated in earlier experiment, were recruited. Inattentive participants, defined as those providing relationship judgments of exactly 0 or 1 in the Memory Task, were excluded, along with 19 participants who self-reported uncarefully. This process resulted in a final sample of 120 participants included in the analyses (*M* = 19.49 years, *SD* = 1.11 years; 63 females, 57 males). This sample size provided 80% power to detect an effect size of r = 0.18 or greater in a repeated-measures analysis, with a 5% false-positive rate. All participants were right-handed, had normal or corrected vision with no reported color vision deficiencies, and received 5 course credits as compensation.

### Material and procedure

#### Network design

We utilized the igraph in R to generate social networks, employing the G(n, m) algorithm to construct random networks and dividing them into communities with the Girvan-Newman algorithm. To minimize cognitive load, we selected a network comprising 10 nodes and 12 edges, which yielded a modularity score of 0.33—similar to real social networks (see Supplementary B). The experimental setup controls for a single common knowledge (a shared club or negative relationship) to eliminate the effect of unequal knowledge counts (Supplementary B). We used neutral cartoon female faces as stimuli to keep the focus on knowledge dynamics within the network.

#### Experimental paradigm

**Fig. 6:**
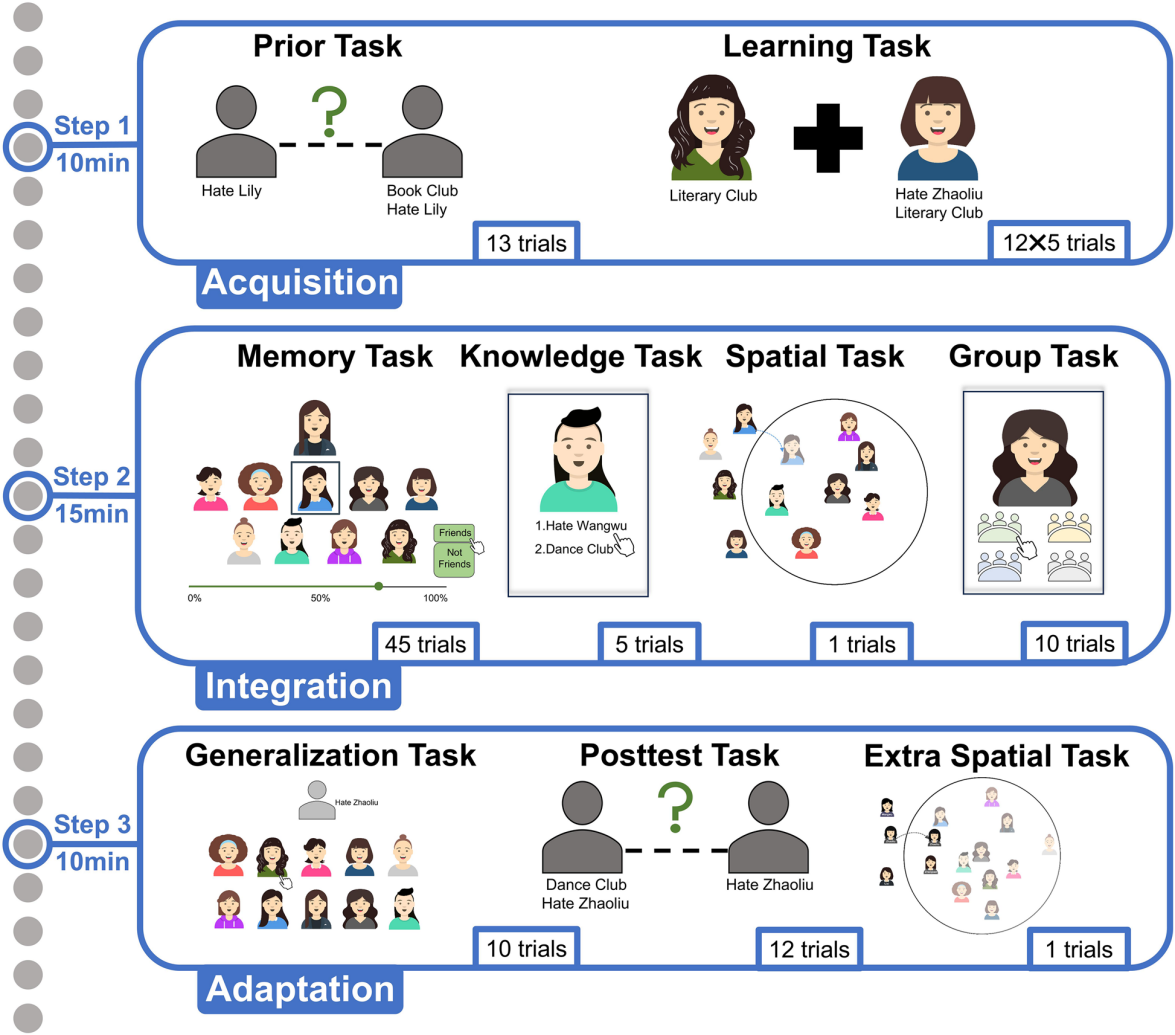
Experiment 5 Paradigm Overview. Note. Participants were told that they would infer relationships based on social knowledge, after which they were shown the true relationship for each pair, including individual-related social knowledge, as shown in Step 1. In Step 2, participants were asked to recall each person’s friendship and social knowledge, and then each person was assigned a position. Finally, in Step 3, participants inferred relationships between strangers and network members and again inferred relationships based on social knowledge before assigning positions to all people

The purpose of the experiment was to investigate how individuals utilize social knowledge within social networks to infer relationships. Experiments adapted from the previous experiment [45], the entire experiment lasted thirty minutes and consisted of nine tasks. (A) Prior Task: Participants viewed images of two targets and inferred friendship probabilities (0–100%) based on their social knowledge. 13 instances were presented, with social knowledge assigned by researchers according to network conditions, such as no social knowledge, shared negative relationships, shared club memberships, and combined negative relationships with shared clubs. (B) Learning Task: Two network members appear side by side to symbolize friendship. Participants viewed images of twelve pairs of friendships within the network to learn its structure and social knowledge about the network members. Each of the twelve pairs of friendship relationships was presented five times, with each presentation lasting four seconds. (C) Memory Task: Participants recalled which members were friends and provided a single rating for each distinct pair (i.e., responses for 55 unique pairs). We computed the response matrix by weighting participants’ binary judgments (Yes/No) based on their confidence levels (0–100%). (D) Knowledge Preference Task: Participants viewed images incorporating both network members and social knowledge. They recalled and selected the social knowledge most relevant to that member, repeating this process 12 times. (E) Spatial Alignment Task: Participants drag 12 network members within a circle according to social distance, simulating the spatial arrangement of network members during the cultural festival. Closer positions indicate a higher likelihood of friendship. (F) Grouping Task: Participants are required to seat 12 network members at different tables to simulate a gathering after the cultural festival, dividing them into Group 1, Group 2, Group 3, and a new group. (G) Generalization Task: Participants inferred which network member a new transfer student was most likely to form a relationship with, considering scenarios in which the transfer student had no social knowledge, shared negative relationships, shared club memberships, or shared both negative relationships and club memberships. (H) Post-test Task: A repetition of the a priori task to measure the stability of participants’ beliefs (0–100%). (I) Extra Spatial Alignment Task: Participants recalled the spatial arrangement of 12 network members from a previous task and then dragged 4 new members into the circle according to their social distance. The detailed process is illustrated in Fig. 6.

#### Questionnaire

This study employed the Relationship Mobility Scale developed and validated by Yuki & Schug [46]. The Relationship Mobility Scale comprises 12 questions rated on a 7-point scale, with a Cronbach’s α of 0.80, designed to assess the degree of relationship mobility as perceived by the participant. In this Experiment, the Cronbach’s α coefficient for the Relationship Mobility Scale was found to be 0.81 (see Supplementary C).

The Social Connection Scale used in this study was the Chinese version adapted by Wu et al. [47]. The Social Connection Scale is a 9-item, 7-point Likert scale that has a Cronbach’s α of 0.92 and a test–retest reliability of 0.85. It measures an individual’s subjective perception of their ability to maintain close interpersonal relationships within their community. In this Experiment, the Cronbach’s α coefficient for the Social Connection Scale was found to be 0.90 (see Supplementary C).

## Result and discussion

To determine participants’ a priori beliefs before acquiring social knowledge, the linear regression results from the prior task indicated significant effects of positive characteristics (*β* = 0.14, *SE* = 0.02,* t* (1557) = 6.35, *p* < 0.001) and negative relationships (*β* = 0.12, *SE* = 0.02,* t* (1557) = 6.35, *p* < 0.001) on the probability of friendship. This suggests that participants utilize social knowledge to infer friendship even before being exposed to the network.

### Representational Similarity Analysis (RSA) Result

**Fig. 7:**
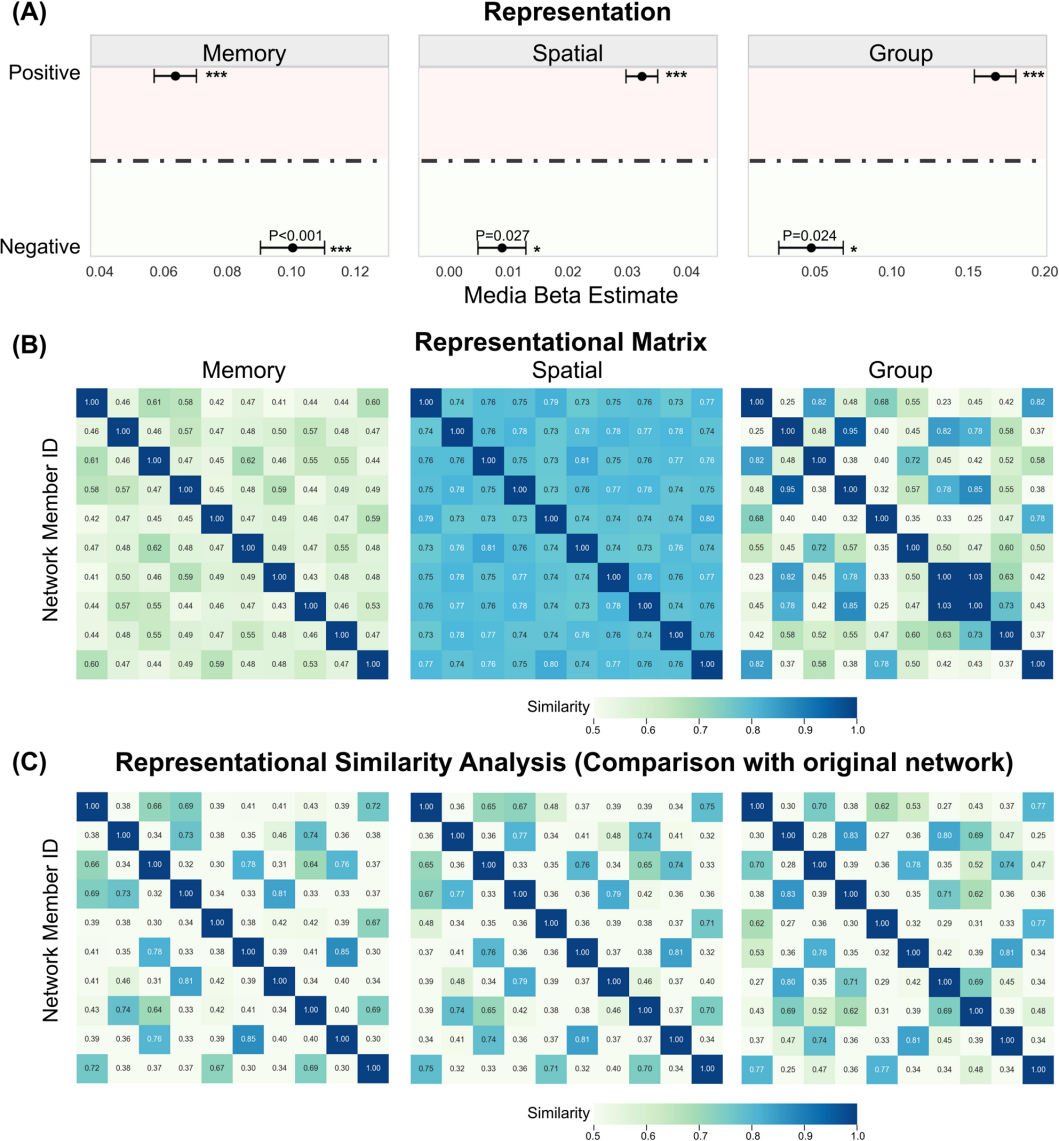
Summary of results from Experiment 5. Note. **A** Forest plots of the three task comparisons, with dots reflecting median beta estimates and error bars reflecting 95% CI. **B** Participants’ response matrices. For the memory task, we constructed response matrices by weighting memories according to confidence levels. For the spatial task, we generated response matrices based on pairwise Euclidean distances, 1 = Maximum similarity (friendship). For the group task, response matrices were created using group assignments. **C** Characterization of similarity analysis methods, similarity between participants’ response matrices and network raw matrices

We conducted the pre-registered RSA analysis in Python 3.11. Participants’ representations were measured by the mean estimates from the memory task, spatial alignment task, and group task. In the memory task, positive characteristics (*β* = 0.06, *SE* = 0.01, *t* (5278) = 9.62, *p* < 0.001) and negative relationships (*β* = 0.10, *SE* = 0.001,* t* (5278) = 10.05, *p* < 0.001) had significant effects. In the Spatial Alignment Task, positive characteristics (*β* = 0.03, *SE* = 0.003, *t* (5278) = 12.15, *p* < 0.001) and negative relationships (*β* = 0.01, *SE* = 0.004, *t* (5278) = 2.21, *p* = 0.027) also exhibited significant effects. In the group task, positive characteristics (*β* = 0.17, *SE* = 0.01, *t* (5278) = 12.40, *p* < 0.001) and negative relationships (*β* = 0.05, *SE* = 0.02, *t* (5278) = 2.26,* p* = 0.024) demonstrated significant effects as well. Overall, participants form social knowledge representations that vary across tasks (Fig. 7A). This disparity likely stems from task-specific influences on decision-making strategies. In spatial alignment and memory tasks, negative relationships may be used to enhance memory and self-confidence. Conversely, positive characteristics are more activated, biasing choices toward ingroup cooperation in group task.

To gain a deeper understanding of these phenomena, we conducted an RSA. The Pearson correlation coefficient, which quantifies the difference between task representations and the original network, was highest for the memory task (*r* = 0.70). The spatial alignment task (*r* = 0.55) and the group task (*r* = 0.57) also exhibited moderately strong correlations. These findings suggest that the acquisition of social knowledge led to varying degrees of shifts in the participants’ representations (Fig. 7B C).

### Generalization and post-test task

We investigated whether participants relied on cognitive maps constructed from prior experiences and acquired knowledge to make relational inferences without having met the new member. Results from regression analyses on the generalization task indicated a significant effect of social knowledge, with the most substantial effect attributed to positive characteristics (*β* = 0.10, *SE* = 0.01, *t* (1006) = 18.07, *p* < 0.001). This was followed by the interaction of positive characteristics and negative relationships (*β* = 0.08, *SE* = 0.01, *t* (1006) = 11.99, *p* < 0.001), and the effect of negative relationships alone (*β* = 0.03, *SE* = 0.001,* t* (1006) = 3.46, *p* < 0.001). This phenomenon aligns with existing theories of cognitive and social reasoning, emphasizing that humans tend to rely not only on direct experiences when understanding social dynamics but also on cross-situational reasoning based on an abstract understanding of the social network.

Results from a paired-sample t-test on the pre-test and post-test task indicated that participants’ beliefs changed after acquiring social knowledge (*t* (119) = −3.75, *p* < 0.001). Linear mixed-effects modeling further confirmed this shift, demonstrating participants’ increased reliance on social knowledge after learning about the network, which included both positive characteristics (*β* = 0.22, *SE* = 0.001,* t* (1438) = 22.56, *p* < 0.001) and negative relationships (*β* = 0.17, *SE* = 0.01, *t* (1438) = 16.54, *p* < 0.001). Analysis in conjunction with the post-test task and knowledge preference task revealed that participants preferred learning and utilizing social knowledge. Specifically, participants who favored negative relationships tended to infer friendship between two targets who shared a negative relationship (*β* = 0.18, *SE* = 0.04, *t* (357) = 5.28, *p* < 0.001), while participants who preferred positive characteristics tended to infer friendship between two targets who shared a positive characteristic (*β* = 0.22, *SE* = 0.03,* t* (357) = 7.82,* p* < 0.001).

### Cognitive mechanism

Various cognitive mechanisms are investigated using distinct experimental paradigms. Specifically, the prior task and posttest task are classified as engaging similarity heuristic processes, in which participants infer relationships by drawing upon pre-existing social knowledge. The knowledge preference task and generalization task involve a cognitive map of social knowledge, requiring participants to utilize their memory of social knowledge about network members to reason about the new relationships. Finally, the memory task, spatial alignment task, and grouping task exemplify featureless associative learning, characterized by participants making inferences based on prior experience without relying on social knowledge. Structural equation modeling was conducted using the lavaan package in R to identify the cognitive mechanisms that best explain participants’ representations. The findings indicated that the use of different mechanisms varied for relational inference. The best models combined associative learning (*M* = 0.03, *z* = 9.21, 95%CI [0.03, 0.04], *p* < 0.001), similarity heuristics (*M* = 0.07, *z* = 7.11, 95%CI [0.05, 0.09], *p* < 0.001), and cognitive mapping (*M* = 0.02, *z* = 3.33, 95%CI [0.01, 0.03],* p* < 0.001) (Table 1). The model suggests that individuals rely on the interaction of multiple mechanisms when inferring friendship in social networks, thereby providing a more flexible and comprehensive reasoning mechanism.

**Table 1:** Construct SEM and fit it with maximum likelihood estimation

Mechanism	Loglikelihood	AIC	BIC
Similarity Heuristic	362.06	−714.12	−694.69
Associative Learning	794.30	−1570.61	−1535.63
Cognitive Maps	1021.75	−2033.49	−2009.30
Associative Learning + Similarity Heuristic	1158.44	−2276.89	−2199.17
Associative Learning + Cognitive Maps	978.98	−1917.95	−1846.43
Associative Learning + Similarity Heuristic + Cognitive Maps	1289.72	−2509.43	−2384.28

The larger value of loglikelihood indicates that the model has a high degree of goodness of fit, while the smaller values of Akaike information criterion(AIC) and Bayesian Information Criterion (BIC) indicate that the model avoids over-fitting while controlling the complexity, and the overall fitting effect is good

Further analysis revealed individual differences in cognitive mechanisms within social networks. Specifically, an individual’s relational mobility significantly influenced performance on the post-test task (*β* = −3.42, *SE* = 1.56,* t* (256) = −2.20, *p* = 0.029) and the group task (*β* = −2.30, *SE* = 1.04, *t* (256) = −2.22, *p* = 0.027). In contrast, the degree of social connectedness did not have a significant effect on participants’ task performance. Utilizing multifactorial variable modeling, we identified a negative impact of relational mobility on the associative learning mechanism (*β* = −0.01, *SE* = 0.01, *t* (358) = −2.40,* p* = 0.017) and similarity heuristics mechanism (*β* = −0.01, *SE* = 0.01, *t* (358) = −3.50, *p* < 0.001). However, the cognitive map mechanism was not significantly affected, providing further evidence of the stability of the cognitive map.

**Fig. 8:**
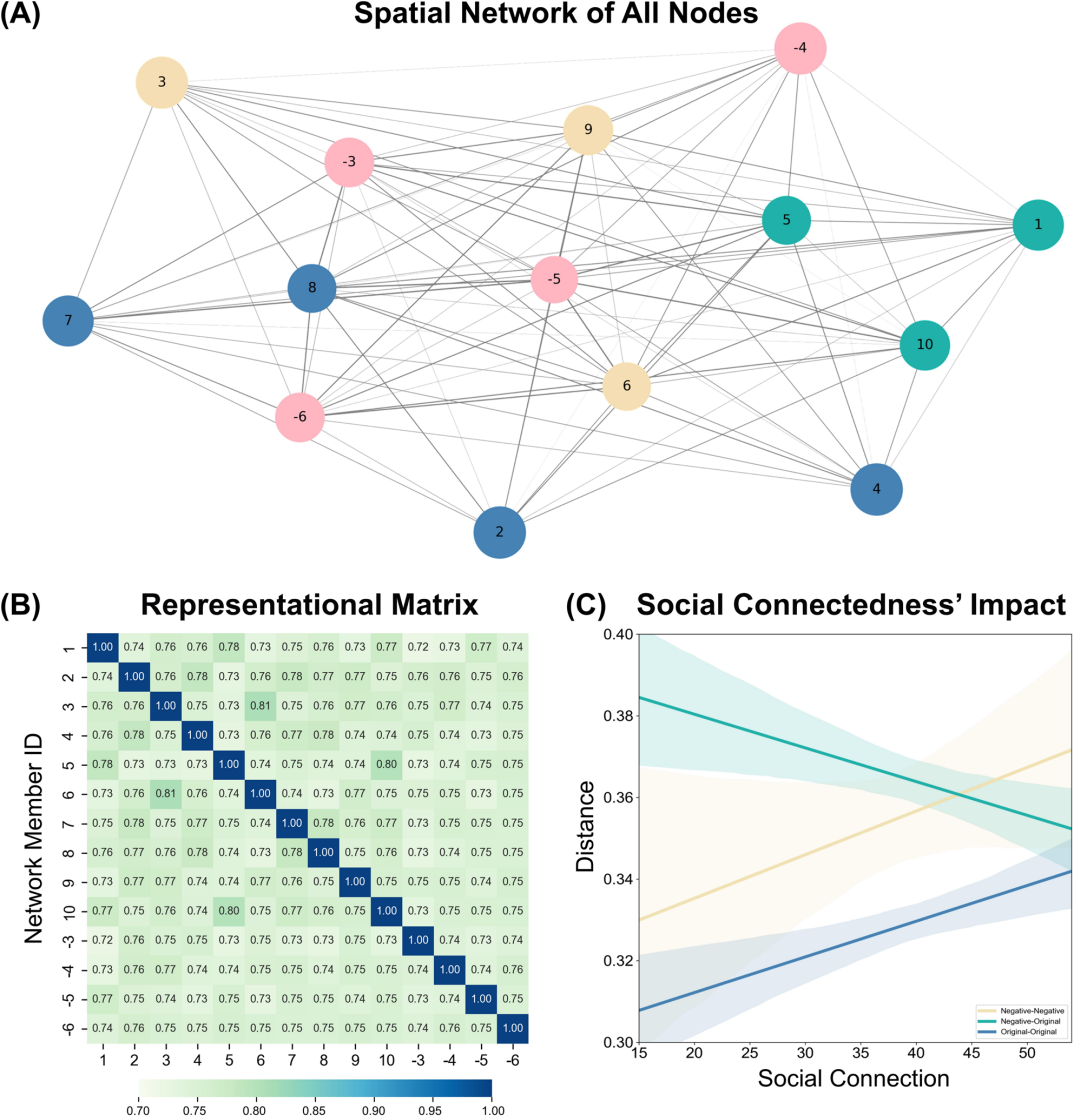
Experiment 5 Summary of results from network analysis. Note. **A** Social network containing original network nodes with negative nodes, nodes of the same color represent being a small group, and the color shade of each line represents the strength of the relationship. **B** Participants’ response matrix containing the original network nodes and negative nodes, with the distance between nodes reflected by pairwise Euclidean distances in the spatial task. **C** The effect of participants’ degree of social connectedness on pairwise Euclidean distances

Network analysis revealed that participants consistently preferred to include negative third-party relationships within the network (Fig. 8A B). We also observed that the distance between negative and original nodes decreased as the degree of social connectedness increased (*β* = −0.004, *SE* = 0.002, *t* (4798) = −2.20, *p* = 0.028). The network showed a weak but notable tendency for individuals to mitigate negative relationships by enhancing connectedness (Fig. 8C). This suggests that social networks’ inclusiveness may extend beyond merely managing negative ties. Such adaptive mechanisms, combined with maximizing group benefits, likely reflect a trend in human societies to promote cohesion and mutual support.

## General discussion

Social knowledge plays a crucial role in enabling humans to infer relationships during social interactions. In five experiments, we examined whether people infer relationships based on shared negative relationships, testing hypotheses related to these associations. We elucidate the instrumental role of shared negative relationships in fostering positive bonds and investigate how the flexible use of social knowledge drives the construction of cognitive maps and shapes representations within complex social networks. Additionally, we examine how intergroup relations shape individuals’ expectations regarding friendship development and highlight the adaptable nature of human cognitive mechanisms in navigating complex social environments.

The principal finding of this study is that individuals tend to infer positive relationships even when the social relationships they share are negative. Prior research has extensively established that similarity functions as a fundamental cue for deducing friendships; for example, common friends, aligned preferences, or shared group identities effectively prompt such inferences [48, 49]. The inferential mechanism based on shared negative relationships has not been systematically investigated. This study posits that shared negative relationships serve as highly informative cues by clearly delineating boundaries between “us” and “them,” thereby simplifying complex social cognitive representations. Drawing on cognitive load theory, this delineation reduces processing demands, lowering cognitive burden and making inference more efficient. This simplification amplifies the propensity to infer positive relationships. This finding aligns with negativity bias theory, which asserts that negative information holds greater significance for survival and decision-making [50]. Accordingly, alliances forged through shared negative experiences may be perceived as more urgent and dependable. Furthermore, results indicate that a greater number of shared negative relationships correlates with a higher probability of inferred friendship. This supports the notion that relational similarity operates as a fundamental mechanism of interpersonal attraction, with effects that may transcend the emotional valence of the relationship itself. Overall, this study broadens the application of negativity bias theory within social cognition by systematically elucidating the critical role of shared negative experiences in the development of social bonds from the perspective of relational inference.

Research demonstrates that the effect of group size on the inference of interpersonal relationships is not fixed but depends on specific social contexts. In Experiments 1 and 2, when two people and a large group share a negative relationship, observers are more likely to infer a friendship between the two people. This phenomenon is grounded in the idea that large groups possess greater social capital, providing more resources and opportunities for relationship formation. Conversely, in contexts characterized by well-defined group boundaries (as examined in Experiments 3 and 4), cognitive processing undergoes a shift. Small outgroups, which possess limited social capital [51] and exhibit heightened visibility of social ties [52], intensify the perceived risks of conflict within negative relationships, leading individuals to adopt more cautious inference strategies. Moreover, clear group boundaries reinforce ingroup identification [53], enhancing individuals’ ability to detect signals of cross-group alliances and thereby improving the accuracy of relational inferences. In sum, this study elucidates a dual mechanism through which group size influences relational inference: in contexts of ambiguous group boundaries, individuals rely predominantly on social capital considerations, whereas in contexts with clear boundaries, reasoning shifts toward identity-based processes. These findings not only corroborate the social relationship model positing that individuals and bilateral relationships are nested within group structures [54] but also underscore the necessity of integrating both interpersonal and intergroup perspectives to develop a comprehensive theoretical framework for social reasoning.

The findings of Experiment 5 align with those of previous research in highlighting the key role of social knowledge in shaping cognitive representations, which reflects the flexibility of human cognitive processing. Individuals often rely on acquired social knowledge to subjectively infer interaction patterns within social networks, rather than depending exclusively on objective memory [7]. This suggests that relational inferences are driven not only by objective reasoning but also by internalized knowledge and cognitive biases. Furthermore, relational mobility moderates how social knowledge is applied. High relational mobility enhances perceived control over social relationships, which has been linked to increased subjective well-being [55]. As a form of relational freedom, it promotes efficient use of social knowledge by encouraging adaptive learning and interaction. In such contexts, individuals tend to rely more on flexible cognitive maps rather than rigid stereotypes, allowing them to navigate dynamic social environments more effectively. Notably, people tend to incorporate shared negative relationships into their social networks, with social connectedness related to their acceptance of these relationships—demonstrating a tendency to conform to the prevailing social context. These findings underscore the role of personal traits and social context in shaping relational inferences and the deployment of cognitive mechanisms.

Based on existing research, we argue that the cognitive significance of negative relationships is due to their adaptive value at both the individual and group levels. By synthesizing theoretical perspectives from interpersonal and intergroup relations, we develop a conceptual framework elucidating the value. Social exchange theory suggests that recognizing this utility entails converting negative perceptions into constructive, actionable knowledge, thereby promoting coordinated commitment [56, 57]. Our findings support this framework: when inferring friendships, individuals tend to prioritize relationship exchanges with positive potential (e.g., shared aversions), thereby avoiding cognitive overload from tracking all relationships and reducing hostility triggered by negative interactions [58, 59]. For instance, political parties enhance overall resilience by exchanging low-value resources [60], while individuals strengthen bonds by sharing aversions toward third parties, thereby fostering positive relationships. Concurrently, network science research indicates that humans exhibit generalization tendencies when recalling social connections, such as categorizing individuals by group [61] and inferring ternary closures [62, 63], while social characteristics effectively predict potential network connections [64]. In contrast to prior research emphasizing the antagonistic nature of negative ties, the present study underscores their functional role in facilitating the prediction of positive network connections. This perspective enhances understanding of how individuals more accurately identify prospective social links, thereby offering novel insights into the cognitive processes underpinning the construction of complex social networks.

Our findings align with classic theories like social cognition, interpersonal relations, and intergroup relations, while also contributing to contemporary social network research, deepening our understanding of social relationships and cognition. Building upon prior research emphasizing humans’ lifelong capacity for “in their own right” social inference [48], it advances toward a more comprehensive cognitive framework. Our results enable more accurate prediction and explanation of the complexities in interpersonal and intergroup relationships, especially within often-overlooked negative relational contexts. We specifically highlight the potential positive significance of negative relationships, revealing their crucial role in shaping positive connections. This opens new avenues for future research to deepen our understanding of relational dynamics and their underlying cognitive mechanisms.

### Limitations and future directions

All research has limitations, and this study is no exception. Our investigation relied on virtual environments without validation in real-world relationships, where social costs and contextual risks could influence the results. Future research should replicate our findings in both offline and virtual reality settings to enhance their validity. We believe that shared relationships offer significant advantages, making it essential to distinguish between positive and negative relationships due to individuals' complex cognitive abilities and sensitivity in social interactions. Therefore, future studies should further explore various social relationships, identifying distinct characteristics and effects associated with different dynamics. Additionally, factors such as group type (e.g., work, family, education) and broader social contexts (e.g., values, religious beliefs) may influence relationship formation and interpersonal interactions. While our findings suggest that social capital and group boundaries explain the varying effects of group size across experiments, these mechanisms require direct testing. Future research should focus on environmental factors and the interplay between group types and settings. Finally, cross-cultural differences should be considered, as they may limit the applicability and generalizability of certain social theories or models.

## Conclusion

Social interactions illuminate the complex nature of interpersonal relationships. Our research demonstrates that shared negative relationships serve as tools for inferring both interpersonal and intergroup dynamics. The inference of friendships based on shared negative relationships is consistently observed across interpersonal, intergroup, and social network contexts, highlighting the enduring significance of such relationships. Furthermore, these inferences are significantly influenced by factors such as the number of relationships and group size. Furthermore, we identified a dynamic process whereby individuals flexibly apply cognitive mechanisms based on subjective experiences to shape their social network representations, exhibiting notable individual differences. This work underscores that human social behavior is deeply intertwined with social cognition and network structures. The inclusivity of social networks may help mitigate the adverse effects of negative relationships, providing a unique advantage in relational inference and promoting more adaptive prosocial behaviors, which holds significant implications for organizational conflict management, team leadership, and school bullying intervention. This research lays the foundation for future studies on predicting social behavior, particularly in the domains of social relationships, group dynamics, and social networks.

## Supplementary Information

Supplementary Material 1.

Supplementary Material 2.

Supplementary Material 3.

## Data Availability

The datasets supporting the conclusions of this article are available in the OSF: https://osf.io/h6msn/overview?view_only=802e562fb05345ea9b7053815d884e85.

## Abbreviations

GEE: Generalized Estimating Equations
SE: Standard Error
M: Mean value
N: Sample size
RSA: Representational Similarity Analysis
AIC: Akaike information criterion
BIC: Bayesian Information Criterion
