# Supplementary material for: Shared enemies as prosocial tool: how to infer positive relationships from negative relationships in social networks
Source: BMC Psychol. 2026 Feb 13;14:367. doi: 10.1186/s40359-026-04098-0 (PMC13005460; doi:10.1186/s40359-026-04098-0)
Supplement: Supplementary file 1 — Supplementary Material 1. [file 40359_2026_4098_MOESM1_ESM.pdf]

**Appendix 1** Test summary of the Gee results of experiment 1-2.

| **Independent** | | **Study 1** | | | | **Study 2** | | | |
|---|---|---|---|---|---|---|---|---|---|
| | | B | SE | Wald | P | B | SE | Wald | P |
| Number of relationships (vs Many ) | Less | -2.33 | 0.08 | 856.14 | 0.000*** | -2.15 | 0.10 | 507.62 | 0.000*** |
| | Middle | -1.10 | 0.05 | 573.36 | 0.000*** | -1.02 | 0.06 | 278.53 | 0.000*** |
| Group size (vs Large) | Small | -0.11 | 0.05 | 5.50 | 0.019* | -0.29 | 0.06 | 23.86 | 0.000*** |
| Number of relationships × Group size | | | | 21.12 | 0.000*** | | | 11.70 | 0.003** |

Note. Statistical significance is indicated by asterisks: * p < .05, * * p < .01, * * p < .001, * * * p < .0001.

**Appendix 2** Test summary of the Gee results of experiment 3-4.

| **Independent** | | **Study 3** | | | | **Study 4** | | | |
|---|---|---|---|---|---|---|---|---|---|
| | | B | SE | Wald | P | B | SE | Wald | P |
| Number of relationships (vs Many ) | Less | -0.75 | 0.09 | 75.64 | 0.000*** | -2.61 | 0.10 | 698.05 | 0.000*** |
| | Middle | -0.69 | 0.06 | 121.22 | 0.000*** | -0.96 | 0.04 | 476.59 | 0.000*** |
| Group size (vs Large) | Small | 0.10 | 0.09 | 112.43 | 0.000*** | 0.09 | 0.04 | 4.56 | 0.033* |
| Number of relationships × Group size | | | | 38.84 | 0.000*** | | | 3.13 | 0.209 |

Note. Statistical significance is indicated by asterisks: * p < .05, * * p < .01, * * p < .001, * * * p < .0001.
