# Supplementary material for: Shared enemies as prosocial tool: how to infer positive relationships from negative relationships in social networks
Source: BMC Psychol. 2026 Feb 13;14:367. doi: 10.1186/s40359-026-04098-0 (PMC13005460; doi:10.1186/s40359-026-04098-0)

Instructions for setting up the network side of Experiment 5

The experimental setup controls for a single common knowledge (e.g., a shared club or negative relationship) to eliminate the effect of unequal knowledge counts. Building on prior research showing that more relationships increase the probability of friendship, we set the number of common knowledge to one to explore how even weaker connections (lower similarity), as demonstrated in Experiments 1–4, are acutely perceived by participants and lead to relational inferences. Thus, in real social networks, our focus shifts from the sheer number of relationships to the influence of shared ties and group membership.

Appendix 1 *Experimental 5 Network Design. Nodes of the same color indicate a club.*

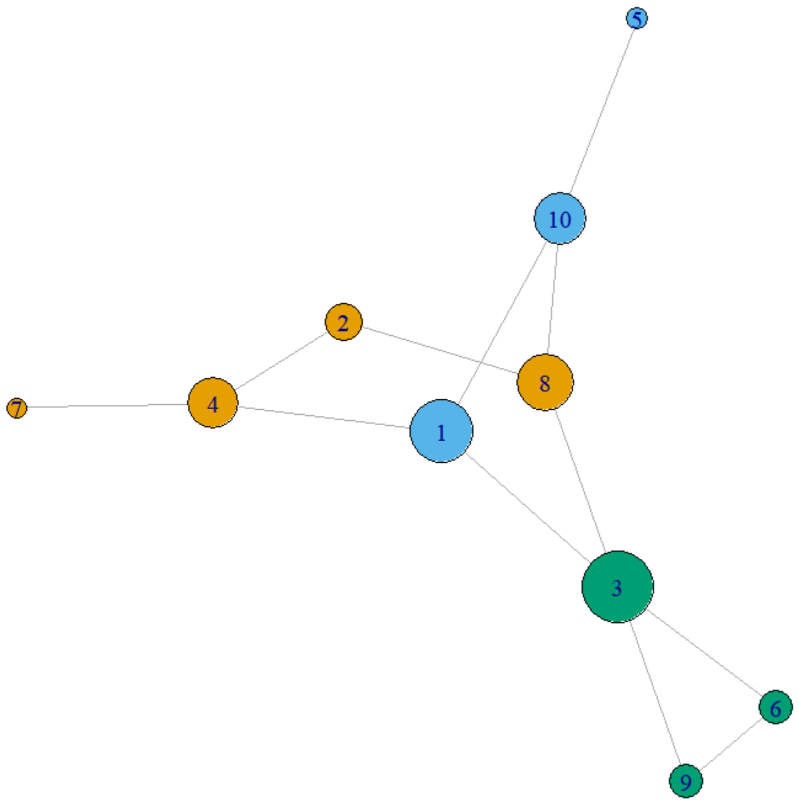

Supplement: Supplementary file 2 — Supplementary Material 2. [file 40359_2026_4098_MOESM2_ESM.pdf]
