# Supplementary material for: Shared enemies as prosocial tool: how to infer positive relationships from negative relationships in social networks
Source: BMC Psychol. 2026 Feb 13;14:367. doi: 10.1186/s40359-026-04098-0 (PMC13005460; doi:10.1186/s40359-026-04098-0)
Supplement: Supplementary file 3 — Supplementary Material 3. [file 40359_2026_4098_MOESM3_ESM.pdf]

Supplementary C

Relational mobility scale in English.

Instruction: How much do each of the following statements accurately describe the people in the immediate society (your school, workplace, town, neighborhood, etc.) in which you live?

Please indicate how true you feel each statement to be for the people around you by circling the appropriate number to the right of each statement.

**Question**	**Strongly Disagree**	**Moderately Disagree**	**Slightly Disagree**	**Slightly Agree**	**Moderately Agree**	**Strongly Agree**
1. They have many chances to get to know other people	**1**	**2**	**3**	**4**	**5**	**6**
2. It is common for these people to have a conversation with someone they have never met before	**1**	**2**	**3**	**4**	**5**	**6**
3. They can choose who they interact with.	**1**	**2**	**3**	**4**	**5**	**6**
4. There are few opportunities for these people to form new friendships	**1**	**2**	**3**	**4**	**5**	**6**
5. It is uncommon for these people to have a conversation with people they have never met before	**1**	**2**	**3**	**4**	**5**	**6**
6. If they did not like their current groups, they would leave for better ones	**1**	**2**	**3**	**4**	**5**	**6**
7. It is often the case that they cannot freely choose who they associate with	**1**	**2**	**3**	**4**	**5**	**6**
8. It is easy for them to meet new people.	**1**	**2**	**3**	**4**	**5**	**6**
9. Even if these people were not completely satisfied with the group they belonged to, they would usually stay with it anyway	**1**	**2**	**3**	**4**	**5**	**6**
10. These people are able to choose the groups and organizations they belong to	**1**	**2**	**3**	**4**	**5**	**6**
11. Even if these people were not satisfied with their current relationships, they would often have no choice but to stay with them	**1**	**2**	**3**	**4**	**5**	**6**

12. Even though they might rather leave, these people often have no choice but to stay in groups they don't like	**1**	**2**	**3**	**4**	**5**	**6**

Note. Items 4, 5, 7, 9, 11, and 12 are reversed.

Social connection scale in English.

Instruction: What is described below is your thoughts and feelings about your relationship with yourself and others. Please read each sentence carefully and choose a number that is most in line with your actual situation and tick "√". "1" stands for "totally disagree" and "6" stands for "totally agree".

**Question**	**Totally disagree**	**Relatively disagree**	**Somewhat disagree**	**Somewhat agree**	**Relatively agree**	**Totally agree**
I feel alienated from others.	1	2	3	4	5	6
I feel irrelevant to most people.	1	2	3	4	5	6
I feel like an outsider.	1	2	3	4	5	6
I feel like an unsociable and lonely person.	1	2	3	4	5	6
I feel isolated from the world around me.	1	2	3	4	5	6
I feel it is difficult for me to blend in with anyone or group around me.	1	2	3	4	5	6
I don't feel a sense of belonging even among familiar people.	1	2	3	4	5	6
I found myself out of touch with society.	1	2	3	4	5	6
I find that I can actively integrate into people's lives.	1	2	3	4	5	6

Note. Except F9 positive scoring, all others are negative scoring. The higher the score, the higher the level of social connection.

Relational mobility scale in Chinese (关系流动性量表中文版).

指导语：下列各项陈述在多大程度上准确描述了你所生活的直接社会环境（如学校、工作场所、城镇、社区等）中的人们？ 请通过圈选每项陈述右侧对应的数字，表明你认为这些陈述对周围人群的真实程度。

**题目**	非常不同意	不同意	稍微不同意	稍微同意	同意	非常同意
1. 他们（你周围的人）有很多机会认识其他人。	1	2	3	4	5	6
2. 对他们来说，和一个素未谋面的人交谈是一件平常的事。	1	2	3	4	5	6
3. 他们可以根据自己的喜好，选择在日常生活中和谁交往。	1	2	3	4	5	6
4. 他们很少机会结交新朋友。	1	2	3	4	5	6
5. 对他们来说，和一个素未谋面的人交谈是不平常的事。	1	2	3	4	5	6
6. 如果他們不喜歡現在身處的群組，他們可以離開，加入更好的群組。	1	2	3	4	5	6
7. 他們時常不能自由選擇和誰交往。	1	2	3	4	5	6
8. 他们很容易可以遇到新相识。	1	2	3	4	5	6
9. 即使这些人对他们所属的群组并不完全满意，他们通常仍会留下。	1	2	3	4	5	6
10. 他们能够选择他们所属的群组和组织。	1	2	3	4	5	6
11. 即使这些人对他们现时的人际关系不满意，他们时常会没有选择地留下。	1	2	3	4	5	6
12. 即使他们宁愿离开，他们通常会没有选择地留在他们不喜欢的群组里。	1	2	3	4	5	6

注：第 4、5、7、9、11 和 12 项为反向计分。

Social connection scale in Chinese (社会联结量表中文版).

指导语：以下描述的是你对自身与他人关系的思考和感受。请仔细阅读每句话，选择最符合你实际情况的数字并勾选“√”。“1”代表“完全不同意”，“6”代表“完全同意”。

**题目**	**完全不同意**	**比较不同意**	**有点不同意**	**有点同意**	**比较同意**	**完全同意**
F1 我感觉自己与他人是疏远的。	1	2	3	4	5	6
F2 我感觉自己与大多数人不相干。	1	2	3	4	5	6
F3 我感觉自己像个局外人。	1	2	3	4	5	6
F4 我感觉自己是个不合群的、孤独的人。	1	2	3	4	5	6
F5 我感觉自己与周围的世界隔绝了。	1	2	3	4	5	6
F6 我感觉自己很难融入周围的任何人或群体。	1	2	3	4	5	6
F7 即使身处熟悉的人之中，我也没有觉得有归属感。	1	2	3	4	5	6
F8 我发现自己与社会失去了联结。	1	2	3	4	5	6
F9 我发现自己能够积极地融入人们的生活中。	1	2	3	4	5	6

注：除 F9 正向计分，其余都是反向计分，分数越高，社会联结水平越高。
